# A Randomised Controlled Study of Low-Dose High-Frequency In-Situ Simulation Training to Improve Newborn Resuscitation

**DOI:** 10.3390/children8121115

**Published:** 2021-12-02

**Authors:** Joanna Haynes, Siren Rettedal, Jeffrey Perlman, Hege Ersdal

**Affiliations:** 1 Department of Anaesthesia, Stavanger University Hospital, 4011 Stavanger, Norway; hege.ersdal@safer.net; 2 Faculty of Health Sciences, University of Stavanger, 4021 Stavanger, Norway; siren.irene.rettedal@sus.no; 3 Department of Paediatrics, Stavanger University Hospital, 4011 Stavanger, Norway; 4 Department of Pediatrics, Weill Cornell Medicine, New York, NY 10065, USA; jmp2007@med.cornell.edu

**Keywords:** in-situ simulation training, low-dose, high-frequency training, booster training, neonatal resuscitation, positive pressure ventilation, skill mastery, neonatal mortality

## Abstract

Positive pressure ventilation of the non-breathing newborn is a critical and time-sensitive intervention, considered to be the cornerstone of resuscitation. Many healthcare providers working in delivery units in high-resource settings have little opportunity to practise this skill in real life, affecting their performance when called upon to resuscitate a newborn. Low-dose, high-frequency simulation training has shown promise in low-resource settings, improving ventilation performance and changing practice in the clinical situation. We performed a randomised controlled study of low-dose, high-frequency simulation training for maintenance of ventilation competence in a multidisciplinary staff in a busy teaching hospital in Norway. We hypothesised that participants training according to a low-dose, high-frequency protocol would perform better than those training as they wished. Our results did not support this, although the majority of protocol participants were unable to achieve training targets. Subgroup analysis comparing no training to at least monthly training did identify a clear benefit to regular simulation practice. Simulated ventilation competence improved significantly for all participants over the course of the study. We conclude that frequent, short, simulation-based training can foster and maintain newborn ventilation skills in a multidisciplinary delivery unit staff in a high-resource setting.

## 1. Introduction

The need for neonatal resuscitation is ubiquitous and often unpredictable. Positive pressure ventilation (PPV) of the non-breathing newborn is the cornerstone of resuscitation. Studies in both high- and low-resource settings suggest that PPV skills are often sub-optimal [1,2]. Simulation training is widely used to prepare healthcare personnel (HCP) to manage this stressful and time-critical event, and is now an integral part of formal neonatal resuscitation programmes [3,4]. Infrequent training (once a year or less) results in deterioration of knowledge and resuscitation skills in particular [5,6]. Simulation-based booster training may maintain skills acquired in formal training programmes [7]. However, optimal training strategies remain unclear, and studies elucidating this issue are urgently required [8].

Low-dose, high-frequency simulation training (LDHFST) training shows promise in promoting retention of acquired skills [9,10]. Studies from low-resource countries have identified LDHFST as an effective means of not only increasing competence in the simulated situation, but also improving skills and changing practice in the clinical situation [11,12].

The extent to which these findings are transferable to a high-resource situation is less well studied, and it may be that training needs of HCPs in this setting differ from those in studies undertaken in low-resource settings. Using a novel neonatal manikin, we randomised HCPs from six different professions allied to the delivery unit in a busy teaching hospital in Norway to train according to a LDHFST protocol or to train as they wished over a nine-month period following an initial educational session.

## 2. Materials and Methods

### 2.1. Study Setting

This study was conducted at Stavanger University Hospital (SUS), Norway. It is the only hospital in the region with both delivery and newborn services, managing approximately 4500 births per annum and providing care for newborns ≥23 weeks’ gestational age (GA). Resuscitation of babies at birth occurs at three sites: the newborn resuscitation room on labour ward, the cesarean section operating theatre and the midwife-run delivery unit. Most HCPs allied to the delivery unit undergo yearly off-site neonatal resuscitation training according to the national guidelines. Additionally, a fortnightly in-situ multidisciplinary team training session is offered to HCPs working on the delivery unit on the day.

Rate of PPV provision at birth is 3.6%, and most neonates are resuscitated by a paediatrician called to attend the delivery [13]. In some unforeseen resuscitations, PPV is initiated by midwifery or anaesthetic staff. Most PPV is provided using a flow-driven T-piece resuscitator (NeoPuff^TM^, Fischer and Paykel, Auckland, New Zealand).

An ongoing research collaboration, Safer Births Bundle SUS, aims to contribute new knowledge on newborn transition and improve the care of newborns on the day of birth. Initiatives include rapid monitoring of newborns’ heart rate using NeoBeat^TM^ (Laerdal Medical, Stavanger, Norway) [14], recording all PPV provided at birth using Laerdal Resuscitation Monitor (Laerdal Medical, Stavanger, Norway) [15], and multidisciplinary ventilation training with a novel neonatal simulator, NeoNatalie Live^TM^ (Laerdal Medical, Stavanger, Norway) [16].

### 2.2. The Neonatal Simulator

NeoNatalie Live is a low-cost newborn simulator, produced with the specific aim of training competence in PPV. Changing simulated lung compliance and variable heart rate linked to ventilation performance allow HCPs to practise management of newborns with differing degrees of birth asphyxia. Real resuscitation data derived from 1237 newborns informs the algorithm guiding the realistic heart rate response according to PPV provided [17]. An active electrocardiogram allows monitoring of heart rate using the dry-electrode technology NeoBeat, replicating practice in the clinical situation. A sensor measures air pressure in the upper airway. Head-tilt detection identifies upper airway closure due to poor positioning. A cry-sound indicates spontaneous respiration and successful resuscitation. Communication with a training application (NeoNatalie Live, Laerdal Global Health, Stavanger, Norway) allows HCPs to review their performance and the App gives targeted feedback to improve skills in any of four scenarios of increasing difficulty. Bluetooth^®^ technology allows collection of training data in a web-log.

### 2.3. The Study

A prospective, randomised controlled study of the effects of LDHFST on competence in neonatal PPV was performed between April 2019 and April 2021. Approximately 300 HCPs may potentially be involved in neonatal resuscitation. All those working in >50% employment were eligible to participate and invited to give informed, written consent. On enrolment, baseline knowledge and simulated performance of resuscitation (test 1 = T1) were documented using NeoNatalie Live scenario 1 (S1; apnoea, normal lung compliance and compensated heart rate) and scenario 4 (S4; apnoea with low initial lung compliance and decompensated heart rate). Participants were invited to attend a 120- to 180-min personalised education session, including PPV training according to Norwegian neonatal resuscitation guidelines and instruction in the use of the simulator [18]. Sessions were concluded when each individual participant had demonstrated providing effective PPV and felt confident in their ability to train independently with NeoNatalie Live. On completing the educational session, a second documentation of performance (test 2 = T2) was undertaken, repeating S1 and S4. Participants were then immediately randomised into one of two groups, (1) train twice a month or (2) train as often as desired, over a nine-month period. Randomisation was performed using a binary randomisation application, RandomOrg (RANDOM.ORG, Dublin, Ireland), and was undertaken concurrently for all HCPs attending the educational session. These participants then trained on their own using any of three NeoNatalie Live simulators, placed in-situ where resuscitation takes place, receiving immediate performance feedback via the application. Each training session was logged, including timelines and objective ventilation data. Knowledge and simulated performance were tested again after nine months (test 3 = T3) using S1 and S4.

### 2.4. Data Collection

Study participants were observed performing S1 and S4 at each of the three test time-points by the same investigator (JH), and scored according to a protocol developed and evaluated in a pilot study. Demonstration of knowledge (by performance) of the initial steps of resuscitation [18] (with potentially 10 points gained) and ventilation skills assessed objectively by the simulator (potentially gaining a further 30 points) gave a maximum of 40 points for each simulation. Skill points were allocated according to achieving adequate face mask seal, generating sufficient but not excessive inflation pressures, appropriate ventilation rate, % valid ventilations, % ventilation fraction, achieving visible chest rise and time to successfully complete the scenario (with better performance resulting in a shorter time to baby-cry).

### 2.5. Data Analysis

Data analysis was undertaken using SPSS (IBM SPSS Statistics for Windows, Version 26.0. Armonk, NY, USA: IBM Corp).

Test scores are summarised for all participants or for subgroups of participants as mean (standard deviation = sd). T3 scores are presented as boxplots. The number of LDHFSTs performed over nine months is presented as a population pyramid. Points lost at T3 are presented as bar charts.

The primary outcome of T3 scores according to randomisation group was analysed using Kruskal-Wallis test, also used for subsequent subgroup analysis according to training frequency.

Secondary outcomes were analysed as follows: comparison of individual test (T1, 2 or 3) scores across professional groups using Kruskal-Wallis tests; all participants’ score change pair-wise from T1 to T3 (reflecting the effect of study participation), from T1 to T2, (the effect of the education session), and from T2 to T3, (the effect of training) using Wilcoxon signed-rank test; analysis of differences in scenario S1 and S4 scores at each test-point (1–3) using Wilcoxon signed-rank test; progression of test scores from T1 to T2 and T3 according to professional group using Friedman’s Anova.

A *p* value < 0.05 was considered statistically significant.

## 3. Results

### 3.1. Participants and CONSORT Flow Chart

**Figure 1:**
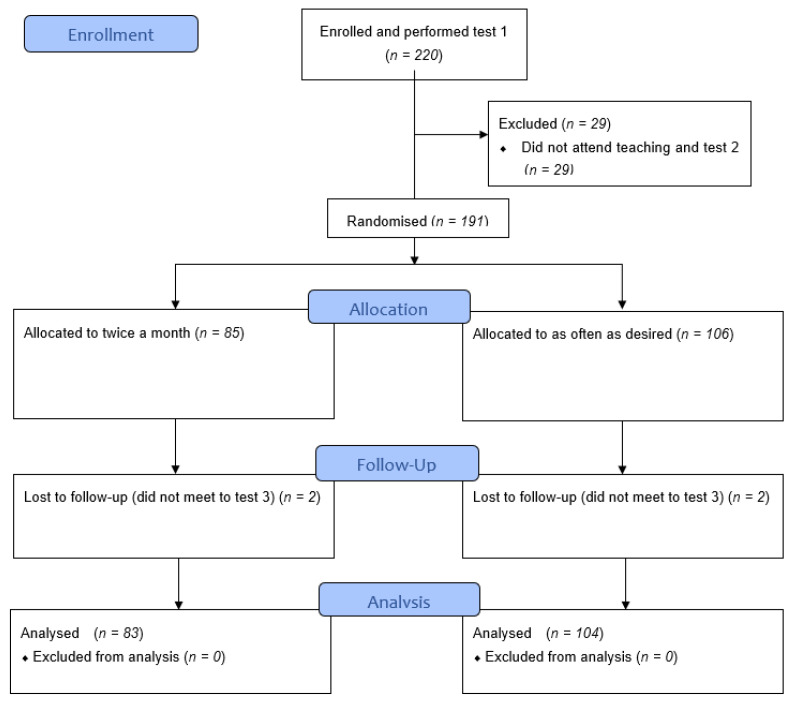
CONSORT flow chart for participants in the randomised controlled study.

220 HCPs were recruited to the study and performed baseline testing, T1. 191 progressed to the education session, performed post-teaching T2, and were randomised. 187 completed nine months of training and performed post-training T3 with four being lost to follow-up (did not meet to test 3). Figure 1 shows the CONSORT flow chart. Table 1 shows the distribution of participants from the six professional groups.

### 3.2. Primary Outcome: Effect of Randomisation Group on Test 3 Scores

**Figure 2:**
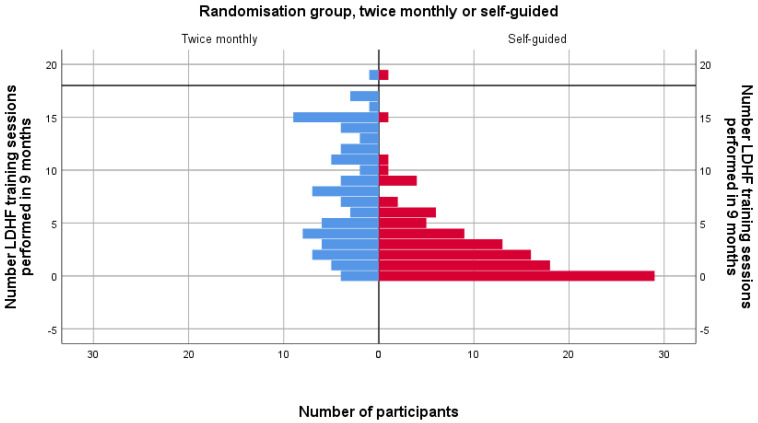
Population pyramid of training frequencies in the two randomisation groups. The black horizontal line indicates 18 trainings (=twice a month). LDHF = low-dose, high-frequency.

Those randomised to train twice a month performed a mean (sd) of 8 (5.2) trainings in nine months while those in the as often as desired (self-guided) trained 2.8 (3.8) times in nine months. Figure 2 shows a population pyramid of training frequencies in the two randomisation groups.

**Figure 3:**
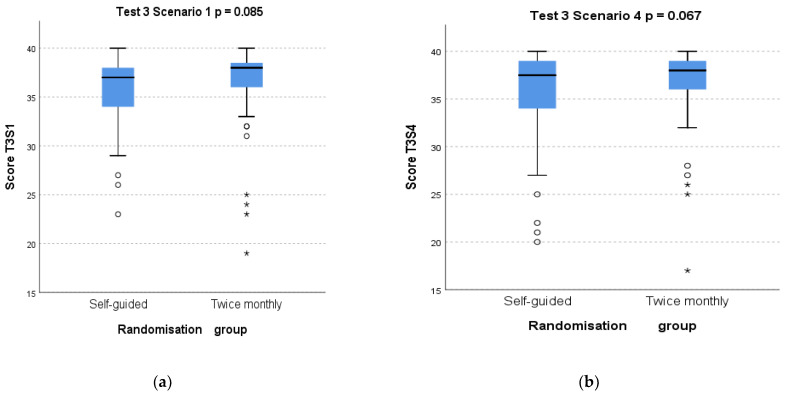
Box plots of test T3 scores for scenario S1 (**a**) and scenario S4 (**b**) according to randomisation group.

T3 scores were not higher in those allocated to train twice a month compared to those training as often as desired, for either S1 (*p* = 0.085) or S4 (*p* = 0.067). Figure 3 show boxplots of T3 scores for both scenarios by training group.

### 3.3. Subgroup Analysis of Test 3 Scores by Training Load

**Figure 4:**
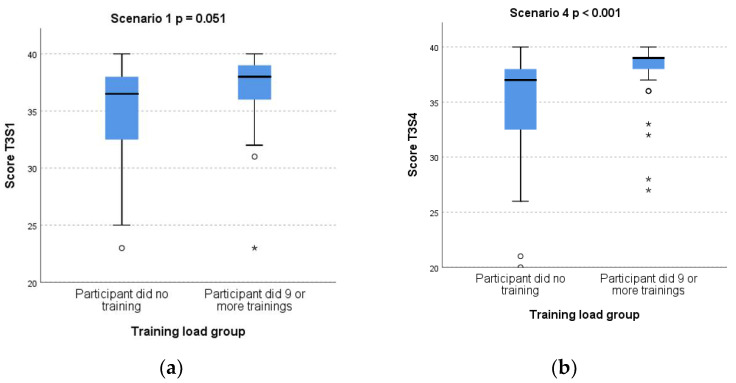
Box plots of test T3 scores for scenario S1 (**a**) and scenario S4 (**b**) according to training-load group.

Subgroup analysis comparing T3 scores for participants performing no training sessions (*n* = 32) and those who performed nine or more trainings (*n* = 43) showed borderline higher S1 score (*p* = 0.051), and significantly higher S4 score, (*p* < 0.001) for those training nine or more times in nine months. Figure 4 shows boxplots of T3 scores for both scenarios by training-load group.

### 3.4. Secondary Outcomes: Effect of Study Participation on Test Scores and Comparison across Professional Groups

**Figure 5:**
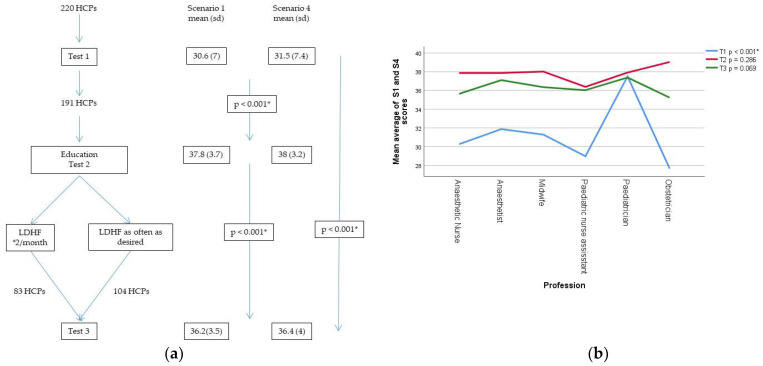
(**a**) Flow diagram of participants through the study with mean (sd) scores for scenario (S)1 and scenario (S)4 of all participants performing each of the three tests; and (**b**) line graph of the professional-group mean of averaged S1 and S4 scores at each test-point. Blue line connects mean T1 scores of the six professional groups, the red and green lines T2 and T3 scores, respectively. HCP = healthcare personnel; LDHF = low-dose high-frequency; * = significant difference in scores at the 0.05 level.

Comparing the average score of both scenarios for all participants, there was an increase in scores from T1 baseline to T2 post-teaching, *p* < 0.001. Following nine months’ training, the mean scores at T3 post-training were lower than T2, *p* < 0.001. Study participation improved mean test scores from T1 to T3, *p* < 0.001. Figure 5a shows the flow diagram of participants through the study along with mean (sd) test scores for all participants performing each test. Analysing this same progression of scores from T1 to T2 to T3 according to profession, this pattern remained the same and the changes were significant for all groups (*p* < 0.001) except the paediatricians (*p* = 0.819). Follow-up analysis of separate scenario scores for all participants at the three test-points demonstrates a higher score for S4 compared to S1 at baseline T1, *p* = 0.014. This was not seen at T2 or T3 where scores for both scenarios were similar.

Analysis of the average score of S1 and S4 at each test 1, 2 and 3 by professional group showed a significant difference only for test 1, in which paediatricians scored higher than other groups; T1 *p* < 0.001, T2 *p* = 0.286, T3 *p* = 0.069. Figure 5b shows these results as a line diagram.

### 3.5. Knowledge and Skills Points Lost at Test 3 by Randomisation and by Training Load

**Figure 6:**
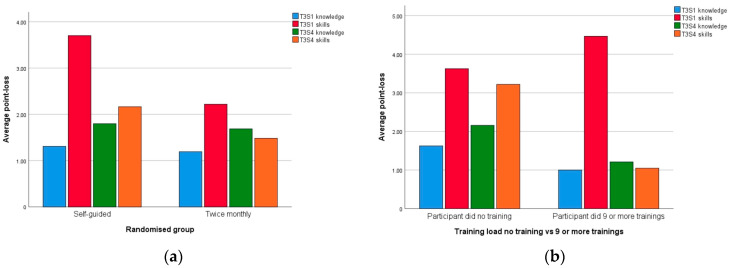
Bar charts of knowledge and skills points lost at test 3 scenarios 1 and 4 (**a**) according to group randomised to and (**b**) according to training-load group. T = test, S = scenario.

Figure 6 shows bar charts of points lost for knowledge and skills in both scenarios at T3 (a) for randomisation groups and (b) training-load groups (subgroups ≥ 9 vs. 0 training). For both comparisons (randomisation and training load), there is a reduction in both knowledge- and skill-point loss when more training is compared to less training, with the exception of S1 skill-points in the nine or more training-load group.

## 4. Discussion

Our randomised controlled study of LDHFST for maintenance of competence in neonatal PPV did not identify improved post-nine months’ training T3 scores compared to participants who trained as desired. However, the twice-monthly group did not achieve the 18 trainings specified in the protocol, performing on average less than 50% of this target in nine months. Subgroup analysis comparing T3 scores of those performing no training with those training at least monthly did identify an advantage to frequent, short simulation training. Competence scores improved significantly over the course of the study for all participants, with analysis by profession identifying the paediatricians as the only group not following this pattern. The paediatricians scored highly from baseline T1. Participation in the study resulted in the scores of all other professional groups improving to the level of the paediatricians at T2 and T3. Regular simulation training improved both knowledge and skills scores.

We studied HCPs coming from six different professional groups. Only one group, the paediatricians, have regular hands-on experience of ventilating newborns at birth. It is unsurprising, therefore, that this group scored significantly higher than the other five at baseline testing T1 on study inclusion. An interesting finding was that a clear learning effect existed on first use of NeoNatalie Live. For all participants, scores for the more demanding S4 were higher than those for S1, which was the first to be performed. This might at first glance seem counter-intuitive. We speculate this reflects the culture for using simulation training in teaching neonatal resuscitation at our institution, with previous experience allowing a rapid adjustment to a new simulator on the first encounter.

Simulation training for enhancing neonatal resuscitation skills has been established as an effective teaching modality [19,20]. Studies investigating simulation training as part of a formal neonatal resuscitation programme have identified learning benefits for birth attendants in low-resource settings and for multi-disciplinary participants in high-resource settings [21,22,23]. Our study findings of improved PPV competence scores at T2 after the educational session echo this. Whilst a limited number of these studies evaluate differences in training benefit between professional groups, our findings highlight the potential for HCPs with little real-life hands-on experience of neonatal PPV to attain knowledge and skills-scores comparable to those with greater experience.

The issue of deterioration of knowledge, and in particular, skills, following formal education programmes is widely acknowledged [6,24,25]. Studies have identified skill deterioration as early as two to three months post-education [26,27,28]. Additionally, the very heterogeneous literature on the effect of booster training strategies to mitigate this deterioration provides conflicting results. A recent systematic review of spaced learning, including booster training, compared to massed learning in resuscitation supported improved performance with spaced learning, but noted that the evidence base was weak and study heterogeneity prevented any meta-analysis [29]. Guideline-issuing authorities have identified the need for studies increasing the knowledge pool on which strategies are effective as a priority [30].

We chose to evaluate a LDHFST strategy, the pedagogical principles of which are established [31,32]. This approach has been shown to maintain simulated skills, contribute to improved clinical performance and to maintain PPV skills in real life in low-resource settings [12,33,34]. One study identifies reduced neonatal mortality in such a setting [11], while another projects reduced mortality with on-going simulation-based performance improvements [35]. The literature on LDHFST and neonatal resuscitation in high-resource settings is sparse by comparison.

Our study participants were randomised consecutively following completion of the education session and performance of test two. This method resulted in an uneven split of participants between the two randomisation groups, although the split by professional group is quite consistent, with only the anaesthetists having more in the <twice monthly> group. Loss to follow up prior to T3 was evenly split between the two groups.

We found that despite the simulator being readily available in the place of work, and clear instructions regarding a non-prescriptive approach to time spent versus validity of training sessions, almost all those in the twice monthly group were unable to achieve this aim. Reasons for this were identified as a heavy clinical workload and the occurrence of a global pandemic during the study period, resulting in a leadership-led de-prioritisation of simulation training for staff. Additionally, those in the <self-guided> group performed on average approximately a third of the number of training sessions achieved by the <twice a month> group. We speculate that our results indicating lack of benefit of our LDHFST protocol result from the fact that we compared two groups who both did some training, where the break point for optimal training load is, as yet, unclear. Subgroup analysis comparing <some> training with <no> training did demonstrate a benefit. This benefit was highly significant for the more complex and demanding scenario 4 (Figure 4b). Another randomised simulation study conducted in the United States found maintained neonatal ventilation skills in those performing booster training monthly or every three months compared to none, but with no difference between the two booster training frequencies [36]. It remains to be determined at what training frequency or training load benefit arises

Whilst study participation did improve competence scores between T1 and T3, the greatest benefits were seen after the educational session, as identified by T2 scores (Figure 5a). It is interesting to consider that one educational initiative is able to improve the performance of HCPs with widely differing backgrounds and clinical experience to the same level. We believe this relates to the personalised approach to teaching, given for the most singly or in pairs, and never exceeding five participants, often from the same professional group. This allowed tailoring of education to the specific needs and clinical role of the participant. For the participants as a whole, scores deteriorated between T2 and T3, although the reduction was modest (Figure 5a). Despite this small score reduction between T2 and T3, we believe our results suggest that a regime of frequent, short, feedback-guided simulation sessions maintain competence gained in education, particularly in light of the fact that T3 scores were not different between the professional groups despite wide variation in real-life PPV experience (Figure 5b). We can, however, only comment on this effect up to nine months post education. We consider it probable that instructor-led, formal education sessions will be necessary at certain longer-term intervals to prevent deterioration that might otherwise occur with prolonged self-guided training.

Previous studies have highlighted that skills deteriorate more quickly than knowledge [36]. Comparing loss of knowledge- and skill-points at T3 shown in Figure 6, there was a reduction in point loss (and thus improved scores) for both knowledge and skills with increased training frequency, both when comparing by randomization and by training-load. The scoring system used in this study is heavily weighted towards objective measures of ventilation skill, and knowledge retention in our data is not comparable to the often extensive testing of knowledge performed in studies using assessments based on the Neonatal Resuscitation Program [37]. On the other hand, our highly objective and detailed assessment of ventilation skills may be a more reliable and valid measurement of PPV competence than that obtained from check-list assessments commonly employed [38]. We hypothesise that the rather unexpected finding of greater skills point-loss at T3 S1 for <nine or more trainings> compared to <no training> reflects the detailed nature of our skills evaluation.

The strengths of this study include the randomised controlled design, allowing comparison of differing training frequencies. Our method of assessment provides an objective and detailed evaluation of ventilation competence. We also studied multidisciplinary HCPs, constituting a majority of the target group for improved training strategies. Weaknesses include an uneven randomisation to the two groups, and the failure of most twice-monthly-randomised participants to achieve the protocol training frequency.

Future studies will use the considerable volume of training data (>2600 simulations) to try to answer the question of optimum LDHFST frequencies to maintain PPV competency, including stratification according to profession. We will also evaluate the details of which aspects of the skill of PPV resulted in greatest point-loss in order to promote targeted training strategies.

## 5. Conclusions

Simulation training with NeoNatalie Live improves PPV competence in multidisciplinary HCPs working in our delivery unit. LDHFST as a booster training strategy after formal instructor-led education successfully prevents skill deterioration. The optimal LDHFST frequency and the optimal interval between formal instructor-led education sessions remain unclear.

## Figures and Tables

**Table 1:** Participants from six professional groups and their progression through the study.

		Total Recruited and Completed Test 1	Educated and Completed Test 2	Randomised to Twice a Month (of Which x Did not Complete Test 3)	Randomised to as often as Desired (of Which x did not Complete Test 3)	Final Total Completing Study and Analysed after Test 3
**Profession**	Anaesthesia nurse	54	46	20 (0)	26 (0)	46
	Anaesthetist	38	34	19 (0)	15 (0)	34
	Midwife	72	62	28 (0)	34 (2)	60
	Paediatric nurse assistant	17	17	6 (0)	11 (0)	17
	Paediatrician	18	18	7 (1)	11 (0)	17
	Obstetrician	21	14	5 (1)	9 (0)	13
Total		220	191	85 (2)	106 (2)	187

## Data Availability

The data presented in this study are available on request from the corresponding author. The data are not publicly available due to privacy statements made in informed consent obtained from participating healthcare personnel.
